# Etiology and pathogenicity of bacterial isolates: a cross sectional study among diarrheal children below five years in central regions of Kenya

**DOI:** 10.11604/pamj.2018.31.88.15644

**Published:** 2018-10-04

**Authors:** Oliver Waithaka Mbuthia, Scholastica Gatwiri Mathenge, Micah Ongeri Oyaro, Musa Otieno Ng'ayo

**Affiliations:** 1 Medical Laboratory Science Department, Kenyatta University, Nairobi, Kenya; 2 Human Pathology Department, Immunology Unit, University of Nairobi, Nairobi Kenya; 3 Centre of Microbiology Research, Kenya Medical Research Institute, Nairobi, Kenya

**Keywords:** Diarrhea, bacteria, enterobacteriaceae, virulence, pathogenicity, prevalence, incidence, diversity, cross-sectional

## Abstract

**Introduction:**

Bacterial agents are among pathogens implicated to cause diarrhea in children resulting to huge mortality and morbidities. Bacterial etiologies causing diarrhea in children below five years are rarely investigated in Central Kenya, which would otherwise guide prescription and target health education.

**Methods:**

A cross-sectional study approach was applied on 163 randomly selected stool samples from children below five years who presented with diarrhea in Murang`a and Muriranja`s hospitals. The objective was to determine the bacterial agents of diarrhea. Enteric bacterial pathogens were cultured using appropriate media and identified. Statistical analyses were performed using STATA v.13. Chi-square or Fisher exact-test were used to check for evidence of relationship whenever applicable.

**Results:**

There were nearly equal distributions in gender 86 (52.8%) female vs. 77 (47.2%) male, majority (35.6%) aged between 0-12 months. Bacterial isolates were highly diverse in female than the male, children aged 49-60 months and least among those aged 0-12 months. A total of 188 bacterial isolates belonging to 11 genera were recovered. The predominant bacteria was nonpathogenic Escherichia coli 85 (45.2%), while 13 (6.9%) Escherichia coli were positive for virulence genes, including 8 (4.3%) positive for LT and STp Shiga-like or Enterotoxigenic Escherichia coli, 3 (1.6%) positive for eae and bfpA Enteropathogenic Escherichia coli and 2 (1.1%) positive for Enteroaggregative Escherichia coli gene. Others included: Salmonella 21 (11.2%), Pseudomonas 14 (7.4%), Shigella 14 (7.4%), Klebsiella 12 (6.4%), Aeromonas 8 (4.3%), Enterobacter 7 (3.7%), Proteus 8 (4.3%), Citrobactor 3 (1.6%), Yersinia 2 (1.1%) and Vibrio 1 (0.5%).

**Conclusion:**

*Salmonella* was the major bacterial isolate and majority of the bacteria were statistically significant cause of diarrhea (p=0.001).

## Introduction

Diarrhea is having loose or watery stools at least three times per day or more frequently than normal for an individual [1]. Despite the efforts in controlling mortality, 9% of all deaths among children below 5 years globally in 2015 were due to diarrhea [2]. This burden of the disease remains unacceptably high. The Millennium Development Goals (MDGs) called for a reduction of child mortality by two thirds between 1990 and 2015. The newly launched and adopted 2030 agenda for Sustainable Development Goals (SDGs) aims to achieve what was not accomplished by MDGs [3]. Globally, diarrhea kills 2,195 children every day, more than AIDS, malaria and measles combined [4]. In Sub-Saharan Africa (SSA), the etiology of diarrhea is seldom known due to the lack of infrastructure for diagnosis. Diarrheal diseases cause 16% of deaths among children below five years in Kenya [5]. Every Kenyan child below the age of five experiences an average of three bouts of diarrhea every year [6]. Under vision 2030, Kenya has committed herself to reduce child mortality by two third among children below 5 years. The prevalence of diarrhea among children below five years in central Kenya stands at 10.4% and 12.1% in Murang`a County [7]. Diarrhea has a myriad of bacterial strains associated with it. The major bacterial pathogens include Escherichia coli, Shigella, Campylobacter, Salmonella, and Vibrio species transmitted mainly through fecal-oral route [1] although other enterobacteriaceae have been linked to cause diarrhea. It is crucial, therefore, to accurately identify the frequency of the broad range of bacterial diarrheal pathogens as well as their virulence genes to better understand bacterial diversity and pathogenicity. Limited continuous surveillance of bacterial etiologies has led to the narrowing arsenal of antibiotic use posing a devastating threat in treatment and management of bacterial-associated diarrhea.

## Methods

**Study site:** The study was carried out in Murang`a North located in Murang`a County, Kenya. Study sites included the two major hospitals; Muranga`a County Referral Hospital and Muriranja`s Level 4 hospital.

**Research design:** A cross-sectional study approach was used in this study.

**Target population:** The research assessed children up to five years of age who reported cases of diarrhea from either of the two hospitals.

**Sampling design:** Sample selection was done using the systematic random sampling where the first unit (case) was selected randomly in each hospital. The n^th^ case after the starting point followed a systematic selection. The n^th^ case represents the sampling interval which was calculated by dividing the approximate total number of diarrhea cases by the sample size of 163 per facility. Therefore, every 4^th^ case of diarrhea (Muriranja`s hospital) and 5th (Murang`a Hospital) were selected until a sample size of 163 was reached from both hospitals.

**Sample size determination:** Applying the formula for estimating the population proportion with specified relative precision described by Daniel 1999 [8] setting the α at 0.05, and a detection rate of 12.1% for children below five years infected with diarrheal disease in Murang`a County [7], a total of 163 children were recruited to achieve 0.95 power.

**Data collection instruments:** The procedure that was used in data collection included structured data collection instruments that involved administering questionnaires directed to child`s caretaker and laboratory request forms.

**Validity:** Pre-testing was conducted in the two hospitals prior to validate the research methods and tools. Controls were run whenever necessary.

**Sample collection:** Diarrheal stool samples were collected on the day of presentation at the Hospitals using well labelled sterile leak-proof polypots. Stool appearance was recorded in the Laboratory on the study questionnaire entries and request forms that matched the specimen identification number and then cultured in Cary Blair medium (Oxoid, United Kingdom) which was then properly sealed, labeled and stored at 4-8^º^C for one day. Samples were disposed as per standard operating procedure of infectious material. On the 2^nd^ day, the cultured transport media was put in a cool box with frozen ice packs and shipped within 3 hours to the Kenya Medical Research Institute (KEMRI), Center for Microbiology Research in Nairobi.

### Bacterial culture, isolation and identification

***Escherichia coli (E. coli):*** *E. coli* species were detected according to the methods described in the Bacteriological Analytical Manual (BAM) [9]. 25g of sample was weighed into 225mL of Brain Heart Infusion (BHI) broth with a dilution factor of 1:10 and incubated briefly at room temperature (RT) shaking periodically then allowing sample to settle. The medium was decanted into a separate sterile bottle and incubated at 35^º^C for 3 hours. The content was then transferred into a sterile container containing 225mL double strength tryptone phosphate broth and incubated for 20 hours at 44^º^C. A loop-ful of the broth was then streaked on pre-incubated MacConkey agar plate and incubated for 18-20 hours at 35 ^º^C. Morphology and biochemical characteristics was followed as described by Feng *et al.* [9].

**Molecular identification of DEC:** The *E. coli* pathotypes were determined using multiplex polymerase chain reaction (PCR) with the primer sets in Table 1 [10-15]. The first PCR of this multiplex contained M1 primers for amplification of *eae, bfpA*, VT, and *aggR* genes for identification of shiga toxin-producing E. coli (STEC), enteropathogenic *E. coli* (EPEC), and enteroaggregative E. coli (EAEC) pathotypes. The second PCR contained M2 primers for amplification of LT, ST, *daaE, ipaH,* and *virF* gene targets for identification of enterotoxigenic *E. coli* (ETEC), diarrheagenic *E. coli* (DAEC), and enteroinvasive *E. coli* (EIEC) pathotypes. About 1μL of genomic DNA was mixed with 24μL of a premade mix containing primers at a 0.2μM final concentration and Platinum Blue PCR SuperMix polymerase (Invitrogen, Carlsbad, CA, USA). The PCR amplification consisted of 2 min at 94^º^C denaturing temperature, followed by 40 cycles of 30 sec at 92^º^C denaturing temperature, 30 sec at 59^º^C annealing temperature, and 30 sec at 72^º^C extension temperature. The PCR products were visualized and recorded under ultraviolet light using a 2% agarose ethidium bromide-stained gel. The verotoxin genes in STEC isolates were analyzed using PCR specific for shiga-like toxin 1 (VT1) and shiga-like toxin 2 (VT2).

**Table 1:** Primers set for amplification of specific genes fragment in E. coli pathotypes

Target	Forward	Reverse	Band	Reference
ETEC –LT	CACACGGAGCTCCTCAGTC	CCCCCAGCCTAGCTTAGTTT	508	[10]
ETEC-ST	GCTAAACCAGTARGGTCT	CCCGGTACARGCAGGATTACAACA	147	[11]
EHEC-Stx1	CAGTTAATGTGGTGGCGAAGG	CACCAGACAATGTAACCGCTG	348	[12]
EHEC-Stx2	ATCCTATTCCCGGGAGTTTACG	GCGTCATCGTATACACAGGAGC	584	[12]
EPEC-eae	CCCGAATTCGGCACAAGCATAAGC	CCCGGATCCGTCTCGCCAGTATTCG	881	[13]
EPEC-bfpA	GGAAGTCCAATTCATGGGGGTAT	GGAATCAGACGCAGACTGGTAGT	300	[13]
EIEC-IpaH	TGGAAAAACTCAGTGCCTCT	CCAGTCCGTAAATTCATTCT	423	[14]
EAEC-aatA	CTGGCGAAAGACTGTATCAT	CAATGTATAGAAATCCGCTGTT	650	[15]
EAEC-aaiC	ATTGTCCTCAGGCATTTCAC	ACGACACCCCTGATAAACAA	215	[13]

LT –heat labile toxin, ST –heat stable toxin, Stx1- shiga like toxin 1, Stx2 -shiga like toxin 2, eae-enteropatognic attachment and effacement, bfpA -bundle forming pilus, IpaH-invasion plasmid antigen H.

***Salmonella species:*** *Salmonella species* were detected according to the methods described in the BAM [16]. About 25g samples were dissolved in about 225mL of sterilized buffered peptone water (BPW), blended, and incubated at 37^º^C for 16-20 hours. About 10mL from the incubated BPW culture was selectively enriched into the 100mL sterilized Selenite Cystine Broth and incubated again at 37^º^C for 24-48 hours. After incubation, 1 loop full inoculum from the selective enrichment culture was streaked onto the pre-incubated Bismuth Sulfiite Agar (BSA) and Xylose Lysine Deoxycholate (XLD) agar plate. Morphological identification followed the description outlined by Wallace *et al.* [16]. Further confirmation of biochemical reactive cultures was done by agglutination test with Salmonella polyvalent (O) somatic antisera as described by Wallace *et al.* [16].

***Shigella species:*** *Shigella species* were detected according to the methods described by Andrews and Jacobson [17]. About 25g sample was aseptically weighed into 225 mL Shigella broth in which 0.5ug/mL novobiocin was incorporated and incubated at 37^º^C for 18-20 hours. One loop full inoculum from the Shigella broth culture was streaked on the pre incubated MacConkey and XLD agar plate and incubated at 37^º^C for 18-24 hours. Then the suspected colonies were identified by their cultural, morphological, and biochemical characteristics as described by Andrews and Jacobson [17].

***Vibrio cholera:*** *Vibrio cholera* was detected following the procedure as described in the BAM [18]. About 25g samples were blended with 225mL sterilized APW and incubated at 37^º^C for 16-18 hours. One loop-ful inoculum from the APW culture was streaked on the pre incubated TCBS and CPC agar plate and incubated at 37^º^C for 24 hours. The suspected colonies were identified by their cultural, morphological, and biochemical characteristics as described by Kaysner and Angelo [18].

***Yersinia species:*** *Yersinia species* was detected following the procedure as described in the BAM [19]. About 25g samples were blended with 225mL of Peptone Sorbitol Bile Broth (PSBB), homogenized and immediately incubated at 10^º^C for 10 days. The enrichment broth was then recovered from the incubator and mixed lightly. Further, one loop full of enrichment was incorporated into 0.1mL 0.5% potassium hydroxide (KOH) in 0.5% saline then mixed lightly. One loop full was then streaked on MacConkey plate and another to Cefsulodin-Irgasan Novobiocin (CIN) plate and both were incubated at 30^º^C for 24-48 hours. Morphological and biochemical identification of *Yesinia enterocoloitica* was performed following procedure described by Weagant and Feng [19].

***Aeromonas species:*** About 25g samples were blended with 225mL sterilized APW and incubated at 37^º^C for 16-18 hours. One loop full of the enriched inoculum from the APW culture was streaked on the pre-incubated Ampicillin Sheep Blood Agar (ASBA) and Xylose deoxycholate citrate agar (XDCA) plate and incubated at 37 ^º^C for 24 hours. Biochemical methods were tested using Aeromonas specific O antiserum [20].

***Klebsiella species:*** *Klebsiella species* was detected following the procedure as described by Cheng *et al.* [21]. Stool samples were inoculated on both MacConkey and Simmons citrate-inositol-tryptophan and bile salts (SCITB) agar media which were then incubated at 37^º^C for 48 hours. Typical colonies that appeared yellow on SCITB were picked for spot indole test and TSI.

**Data analysis and presentation:** Frequency (%), mean, standard deviation, and medium (interquartile ranges at 25% and 27%) were used to describe the qualitative and laboratory parameters. Chi-square or Fisher's exact test were used to test for significance where applicable. Bacterial diversity was determined by **Shannon Weaver Diversity Index** using Microsoft excel. All statistical analyses were performed using STATA v.13 (StataCorp LP, College Station, TX, USA).

**Ethical consideration:** Ethical approval was granted by Kenyatta University Ethics and Research Review Committee {KU/ERC/APPROVAL/VOL.1 (31)}. Research permit was given by the National Commission for Science Technology and Innovation (NACOSTI/P/17/15949/16819).

## Results

**Bacterial etiology of diarrhea among the study population:** Table 2 describes the distribution of bacterial etiological agents of diarrhea in overall, among gender and age group. A total of 188 bacterial isolates belonging to 11 genera were recovered from stool samples of children under investigation. The predominant bacteria from stool samples was nonpathogenic E. coli 85 (45.2%), while 13 (6.9%) E. coli were positive for virulence genes, including 8 (4.3%) positive for both LT and STp Shiga-like or Enterotoxigenic E. coli (ETEC), 3 (1.6%) positive for eae Enteropathogenic E. coli (EPEC) and 2 (1.1%) positive for aatA EAEC gene Enteroaggregative E. coli (EAEC). Others included: *salmonella enteric* 19 (10.1%), *salmonella typhimurium* 2 (1.1%), *pseudomonas aeroginosa* 14 (7.4%), shigella boydii 8 (4.3%), *Shigella sonnei* 6 (3.2%), *klebsiella pneumonia* 8 (16.3%), *klebsiella oxytoca* 4 (2.1%), *aeromonas hydrophila* 5 (2.7%), *aeromonas carviae* 3 (1.6%), *enterobacter aerogenes* 7 (3.7%), *proteus vulgaris* 8 (4.3%), *citrobactor freundii* 3 (1.6%), *yersinia enterocolitica* 2 (1.1%) and *Vibrio cholera* O1 1 (0.5%). Pathogenic bacteria were distributed differently across the gender of the child and age group. Pathogenic bacteria ETEC, EPEC, EAEC, *salmonella, shigella, vibrio, aeromonas and citrobacter* were predominant among the female children (p>0.05). Among age groups EPEC, EAEC, *shigella* and *klebsiella species* were predominant among 0 to 12 month while ETEC, EAEC, and *citrobacter*were predominant among those aged 13 to 24 years (p>0.05) (Table 2).

**Table 2:** Distribution of pathogenic bacteria by gender and age group of the study population

	Overall	Gender		Agegroup (months)				
Bacteria stains	No	Female	Male	0-12	13-24	25-36	37-48	49-60
Non- pathogenic E.coli	85 (45.2)	39 (45.9)	46 (54.1)	31 (36.5)	24 (28.2)	16 (18.8)	8 (9.4)	6 (7.1)
ETEC	8 (4.3)	5 (62.5)	3 (37.5)	3 (37.5)	5 (62.5)	0	0	0
EPEC	3 (1.6)	2 (66.7)	1 (33.3)	2 (66.7)	0	1 (33.3)	0	0
EAEC	1 (1.1)	2 (100)	0	1 (50)	1 (50)	0	0	0
Salmonella enterica	19 (10.1)	12 (63.2)	7 (36.8)	0	10 (52.6)	5 (26.3)	2 (10.5)	2 (10.5)
Salmonella typhimurium	2 (1.1)	2 (100)	0	0	0	0	0	2 (100)
Klebsiella pneumonia	8 (16.3)	4 (50)	4 (50)	6 (75)	0	1 (12.5)	1 (12.5)	0
Klebsiella oxytoca	4 (2.1)	3 (75)	1 (25)	0	2 (50)	0	0	2 (50)
Shigelle sonnei	6 (3.2)	3 (50)	3 (50)	5 (83.3)	0	1 (16.7)	0	0
Shigella boydii	8 (4.3)	5 (62.5)	3 (37.5)	3 (37.5)	5 (62.5)	0	0	0
Vibro cholera	1 (0.5)	1 (100)	0	0	0	0	1 (100)	0
Enterobacter aerogenes	7 (3.7)	2 (28.6)	5 (71.4)	0	2 (28.6)	2 (28.6)	2 (28.6)	1 (14.3)
Proteus vulgaris	8 (4.3)	2 (25)	6 (75)	2 (25)	2 (25)	2 (25)	0	2 (25)
Pseudomonas aeroginosa	14 (7.4)	6 (42.9)	8 (57.1)	4 (28.6)	4 (28.6)	3 (21.4)	2 (14.3)	1 (7.1)
Aeromona hydrophila	5 (2.7)	3 (60)	2 (40)	0	0	0	4 (80)	0
Aeromona caviae	3 (1.6)	1 (33.3)	2 (66.7)	0	0	3 (100)	0	0
Citobacter freundii	3 (1.6)	3 (100)	0	0	2 (66.7)	1 (33.3)	0	0
Xersinia enterocolitica	2 (1.1)	0	2 (100)	1 (50)	0	1 (50)	0	0
								
	**188**	**96 (51.1)**	**92 (48.9)**	**62 (32.9)**	**56 (28.9)**	**37 (19.7)**	**17 (19.1)**	**16 (8.5)**

**Figure 1:**
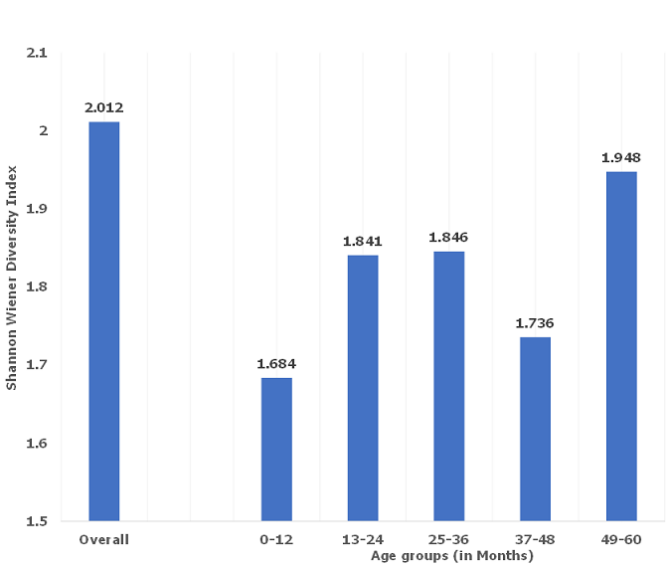
Bacterial diversity using Shannon Weaver Diversity Index across participants' age groups

**Figure 2:**
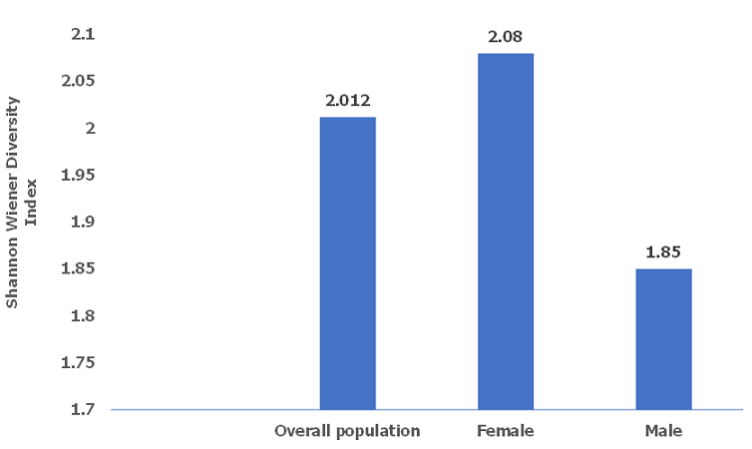
Bacterial diversity using Shannon Weaver Diversity Index across participants' gender

**Bacterial diversity:** Figure 1 and Figure 2 describes bacterial diversity using Shannon Weaver Diversity Index across participants` gender and age groups. Bacterial isolates were highly diverse in female (index=2.08) compared to males (index=1.85). Further, across age, bacteria were more diverse among those aged 49 to 60 (index=1.948) and least among those aged 0 to 12 months (index=1.68).

**Pathogenic escherichia coli virulence genes by PCR:** Table 3 describes distribution of pathogenic E. coli virulence genes by stool appearance. 13 (6.9%) *E. coli* were positive for virulence genes, including 8 (4.3%) positive for both LT and STp Shiga-like or ETEC, 3 (1.6%) for eae EPEC and 2 (1.1%) for EAEC gene.

**Table 3:** Distribution of pathogenic E. coli virulence genes by stool appearance

	PCR types	Stool consistency			
Bacteria strains		watery	Mucoid	Bloody	Water and bloody
*ETEC*	8	6	1	0	1
*LT*	6	4	0	0	2
*STP*	2	0	1	0	2
*EPEC*	3	2	1	0	0
*eae*	2	2	0	0	0
*bfpA*	1	0	1	0	0
*EAEC*	2	1	0	0	1
*aatA*	1	1	0	0	0
*aatA*	1	1	0	0	0
	**13**	**9**	**2**	**0**	**2**

**Distribution of the major pathogenic bacteria by stool appearance:** Table 4 describes the distribution of the major pathogenic bacteria by stool appearance. A total of 188 bacterial strains were isolated from stools of the study participants. Watery stool samples harbored the most bacterial pathogen 65 (34.6%), followed by stool that was both watery & bloody 46 (24.5%) and least from the stool that was only blood stained 36 (19.1%). The type of stool appearance was statistically significant with the isolation of the bacterial strains (p=0.001).

**Table 4:** Distribution of pathogenic E.coli virulence genes by stool appearance

	overall	Stool consistency				
Bacteria stains	No	Watery	Mucoid	Bloody	Watery and bloody	P
*Non- pathogenic E.coli*	85 (45.2)	31 (36.5)	24 (28.2)	16 (18.8)	14 (16.5)	0.001
*ETEC*	8 (16.3)	6 (75)	1 (12.5)	0	1 (12.5)	0.001
*EPEC*	3 (6.1)	2 (66.7)	1 (33.3)	0	0	0.001
*EAEC*	2 (4.1)	1 (50)	0	0	1 (50)	0.001
*Salmonella enterica*	19 (10.1)	4 (21.1)	0	5 (26.3)	10 (52.6)	0.001
*Salmonella typhimurium*	2 (1.1)	0	0	2 (100)	0	0.001
*Klebsiella pneumonia*	8 (16.3)	6 (75)	0	1 (12.5)	1 (12.5)	0.001
*Klebsiella oxytoca*	4 (2.1)	0	2 (50)	2 (50)	0	0.495
*Shigelle sonnei*	6 (3.2)	0	0	1 (16.7)	5 (83.3)	0.001
*Shigella boydii*	8 (4.3)	3 (37.5)	5 (62.5)	0	0	0.001
*Vibro cholera*	1 (0.5)	0	0	0	1 (100)	0.001
*Enterobacter aerogenes*	7 (3.7)	2 (28.6)	2 (28.6)	1 (14.3)	2 (28.6)	0.754
*Proteus vulgaris*	8 (4.3)	2 (25)	2 (25)	4 (50)	0	0.001
*Pseudomonas aeroginosa*	14 (7.4)	4 (28.6)	3 (21.4)	3 (21.4)	4 (28.6)	0.09
*Aeromona hydrophila*	5 (2.7)	0	1 (20)	0	4 (80)	0.001
*Aeromona caviae*	3 (1.6)	3 (100)	0	0	0	0.001
*Citobacter freundii*	3 (1.6)	1 (33.3)	0	1 (33.3)	1 (33.3)	0.001
*yersinia enterocolitica*	2 (1.1)	0	0	0	2 (100)	0.001
	**188**	**65 (34.6)**	**41 (21.8)**	**36 (19.1)**	**46 (24.55)**	

## Discussion

**Bacterial diversity between gender and age groups:** Most studies have evaluated bacterial diversity based on region or locality rather than gender and age groups of children below 5 years. Unlike a study conducted in Kenya [22] that enrolled participants based on geographical diversity across 4 widely distributed localities, the nature of the current study did not allow such comparisons based on geography nor analyze for bacterial diversity on the assumption that participants were drawn randomly from the same locality. The fact that bacterial strains were slightly more diverse among the female than their male counterparts from our study participants may have contributed to the higher bacterial infections associated with diarrhea among the female.

**Diarrheagenic *escherichia coli* (DEC):** The predominant bacteria from stool samples was nonpathogenic *E. coli* 85 (45.2%) lower from what was observed (71%) in Eastleigh among urban refugee children [23]. Our study showed that 13 (6.9%) DEC were positive for virulence genes and these bacteria have been found to be the major bacterial cause of childhood diarrhea especially in developing countries [24]. The finding from this study are broadly in harmony with those of researchers in Angola [25] indicating that DEC accounted for 6.3% prevalence. Colonization by enteric E. coli without eliciting illness is common but their manifestation to cause diarrhea may be attributed to a myriad of factors such as bacterial load, immunity, age, nutrition and environmental factors [26].

**ETEC:** From the study, EHEC was seconded by EPEC as the most often isolate of the DEC types. Notably, 4.3% ETEC were positive for both heat labile toxin (LT) and (heat stable toxin (STp) Shiga-like compared to a study that reported detection of the same ETEC toxins (24.1%) among children in the Maasai community [27] and 38.3% prevalence in Mbagathi hospital [28]. Virulence genes facilitate the bacterial manifestation and pathogenicity. The finding of this study provide evidence that ETEC is a common isolate among children below 5 years within the study region and is a major cause of diarrhea among this group. However, it is worth noting that some of the study participants in our study were drawn from crowded areas where sanitation was not properly observed. Global Epidemiology of ETEC infection documents ETEC as a major bacterial etiology of diarrhea among children below 5 years [29]. Our extract is an interesting example where ETEC isolation dominates other DEC pathotypes among children below 5 years with diarrhea and asserts the strain as a major cause of diarrhea. Relating to previous research, the reason why ETEC was the most common DEC type in our study may have something to do with seasonality, bacterial O groups and H serotype variations and their colonization factors [30]. Evidence of higher ETEC isolation rate during warm seasons with STp Shiga-like ETEC more common during summer [30] may suggest the tendency of higher ETEC isolation in our study. Half of the recruitment of the study participants was done during warm season (August-October). One of the themes that emerged from our study is that all children infected with ETEC were below 2 years suggesting that the immune system improves as the child`s age progresses. Children below 3 years of age are more frequently infected with ETEC with the 1st diarrheal episode more likely to be ETEC [31]. Some studies have shown that the secretory antibodies contained in breast milk protect against diarrhea and hence an assumption that breast fed infants correlates with reduced ETEC induced diarrhea. However, experiments have demonstrated that only temporary protection is offered by bovine colostrum against ETEC challenge [32] and this effect is not seen during the first 3 years [31]. The fact that isolation of ETEC was only below 2 years in this study appears to support this argument.

**EPEC:** Isolation of (1.6%) EPEC possessing eae and bfpA EPEC genes from our study suggests different perceptions to the finding of earlier work that provided a median global prevalence of EPEC at the community (8.8%), outpatient (9.3%) and inpatient (15.6%) among children below 5 years having diarrhea [33]. In several regions, EPEC isolation among children below 5 years has been reported as the major bacterial isolate causing childhood diarrhea, mortality [26] and a cause of outbreak. Our data, however, suggest that other bacterial strains may have a higher affinity of infecting the population hence taking dominance as a cause of diarrhea. The finding that female had a double-fold infection rate with EPEC and a higher infection rate among neonates (< 1 year) are at odds with finding of other researchers [33]. Over 27 eae variants encodes intimin which is fundamental in the attaching and effacing phenotype of E. coli species have been identified [34]. Intimin facilitates attachment of the bacteria to the host cell membrane disrupting the cell surface resulting to effacement of microvilli. We report a lower detection frequency of EPEC genes by multiplex PCR which contradicts previous work in Kenya [27] and China [35].

**EAEC:** In this study, EAEC (1.1%) that harbored the aatA and AggR EAEC gene was the least dominant of DEC types which differs from studies conducted in Kenya [22] and India [36] where this strain was the most dominant isolate among *E. coli* in children below five years. Nonetheless, infants have been found to harbor EAEC more often and 50% of this strain was isolated from children infants (<1 year) while the other 50% affected those between 1-2 years in our study. Interestingly, none of Enteroinvasive E. coli (EIEC) pathotypes was isolated from the study participants despite this type been cited to be a leading cause of profuse diarrhea in some regions.

***Salmonella:*** We isolated more than 3 times *Salmonella species* from what was reported in a Kenyan study [22]. However, our finding are comparable to those of a study done in Meru that reported an isolation rate of 10.4% among children below 5 years [37].The high incidence of *Salmonella* among children below 5 years match the finding shown by researchers in other regions such as India [38] but our finding differs to a greater extent with work done in Ethiopia [39] and Kolkata [36] that shown a lower prevalence of 3.95% and 0.3% respectively. Isolation of Salmonella in children above one year and older children above 2 years in our study suggests that the bacteria is less likely to cause infantile diarrhea harmonizing fairly with finding of a study that observed Salmonella infection was more likely to occur as the child's age progressed [38]. More than half of the Salmonella infections were noted to fall within those aged 13-24 months which conforms with a study done in Lusaka Zambia [40] but sharply contradicts finding of a study in Kumasi Ghana that observed those aged 13-24 months were the least infected (1.5%) while those aged 25-60 months were the most infected [41]. Other than probable food/water-borne Salmonella transmission that may occur during complementary feeding, another possible reason of the data observed that more salmonella infections occurred above one year could be that children of older age interact with domesticated animals such as chicken and other fowl within the households as most participants were drawn from rural and semi-urban areas. Such animals may be the reservoir of salmonella bacteria acting as a principal source. Moreover, maternal antibodies specific to Non Typhoid Salmonella have been proven to be deprived at this age [42].

***Shigella:*** Isolation rate of *Shigella species* overall was 14 (7.5%) running in harmony with finding reported in a previous study that documented an overall *Shigella* prevalence of 7.9% [36]. Isolation rate of *S. boydii* in this study conforms to the global prevalence (4%) of shigellosis cases [43] but none of the *S. flexneri* which has been reported to be the most prevalent sero-group in developing countries was isolated among the study participants. Dominance of specific sero-groups of Shigella may vary depending with multiple factors such as age, sex, comorbidities, geography, sanitation and industrialization. A shift in the etiology of bacillary dysentery by dominance of other *Shigella* sub groups is evident [44]. Probably this observation where previously known major sero-groups that caused infection are been overtaken and the less common sero-groups as observed from our study takes the lead. Our finding closely matches to a greater extent the work reported recently in Ghana [41] that *Shigella species* were generally more common isolates in children below 2 years. Dysentery outbreaks are common especially in developing countries where hygiene and sanitation are questionable. Three (3%) children reported episodes of dysentery 2 weeks prior the Kenya health survey [6]. A much lower isolation rate of 1.4% from what was observed from our study was reported in Beijing [35], probably due to the difference in industrialization.

***Aeromonas:*** *Aeromonas species* accounted for a prevalence of 8(4.3%) from the study population, almost near to what was reported in a study among Eastleigh refugee children below 5 years [23]. A lower prevalence of 2.0% compared to 4.3% from our finding has been reported among children in China [45]. Isolation of *Aeromonas species* was specific only to children between 3-4 years and these finding concur data output of a study done in Pakistan and Bangladesh that documented the bacteria been the leading bacterial pathogen as a cause of diarrhea with a peak between 3-5 years [46].

***Vibrio cholera:*** *Vibrio cholera* species was rare and the least isolate (0.5%) from the study participants despite ongoing outbreaks within the County at the time of the study. Near finding, however, from previous studies (0.7%) in Kenya [22] and (0.4%) elsewhere [35] implicated *V. cholera* as a less prevalent isolate among children up to 5 years. The prevalence may rise during epidemic and *V. cholera* has been associated with severe diarrhea and loss of life. Higher prevalence of up to 40.8% among children less than 5 years in Lusaka Zambia was reported and V. cholera implicated as the most common isolate from diarrheal stool samples [40]. On the available evidence, poor sanitation and contaminated water are the major drivers to the infection [23]. The tendency of toilet training at the age of above 3 years is common which can be hypothesized to elevate fecal oral transmission of this pathogen.

***Yersinia enterocolitica:*** In conformity with the finding of the study, Yersinia species was recovered from stool samples among infants having diarrhea in Denmark [47]. Infants (below 12 months) were 50% infected with *Y. enterocolitica* in this study. Likewise, children between 3-4 years were equally 50% infected which can be related to a study that reported children between 3-4 years were more associated to the bacteria [47]. One possibly that this bacteria was found common among infants below one year could be due to their unchallenged immune system and therefore more likely to be inflicted. Infection by other enteric pathogens elevates the infection by *Y. enterocolitica* as well as domesticated animals such as dogs, cats, pigs and other bovine have been shown to harbor the bacteria [48]. Older children above two years interact more with such animals which may explain the phenomenon that this group were more infected with the bacteria.

***Citrobacter freundii:*** *Citrobacter species* are usually thought to be commensal organisms, though some species have acquired specific virulence genes hence enabling them cause diarrhea. More than double-fold prevalence (3.95%) from the finding of the current study was reported in Addis Ababa, Ethiopia and this bacteria was linked to cause childhood diarrhea [39]. Only the female were found infected with *C. freundii* and those between 1-3 years among study participants. This finding contradicts an observation in a study that showed male (66.7%) were more infected than female (33.3%) [38]. Children who were between 13-24 months had a double fold infection rate with the bacteria than those who were between 25-36 months in this study. Potential factors that may have elevated the infection among this age bracket may point to the exposure of unhygienic and sanitary factors associated with the bacterial infection.

***Klebsiella:*** Male and female participants were equally infected by *K. pneumonia* with majority of the infected below one year (75%) similar to outcome from a different study that observed children below 12 months were more infected with Klebsiella [38]. *K. oxytoca* isolation was less common among the male (25%) and only children between 1-2 years (50%) and those between 4-5 years (50%) were found infected. K. pneumonia and *K. oxytoca* is normal flora of the gut but can cause of diarrhea in human. Experience from other parts of the world has confirmed that *K. pneumonia* was a cause of bloody diarrhea following negative results of other enterobacteriaceae but isolation of *K. pneumonia* from pure colonies was evident [49]. *K. pneumonia* (25%) were isolated from stools that were blood stained and the other 75% isolates were from watery stools (p=0.001). From data output of this study, it is possible to tentatively assume that *K. pneumonia* is a potential cause of diarrhea among the study participants unlike *K. oxytoca* that was found not to have any statistical evidence of significance with the diarrhea (p=0.495).

## Conclusion

Bacterial etiologies are common and are a significant cause of diarrhea among children below five years in Murang`a County, Kenya. Salmonella tyhimurium and S. enterica are the major bacterial agents causing diarrhea among children below 5 years. Not only less attention has been given on isolating and identifying bacteria causing diarrhea among children below 5 years, but also the less common enterobacteriaceae have largely been ignored and these bacteria should be investigated. Data from this study contributes to the current microbial surveillance system in Kenya.

### What is known about this topic

- Enterobacteriaceae are associated with diarrhea;
- Enterobacteriaceae possesses a variety of virulence factors that facilitate in their colonization.

### What this study adds

- Other than the major pathogenic bacteria (Salmonella, Shigella, ETEC, EPEC and EAEC), other less commonly isolated bacteria are strongly significant cause of diarrhea among children below five years;
- Children below one year (neonates) are the major culprits of diarrheal illnesses caused by pathogenic bacteria.
